# Optimization Methodology for Additive Manufacturing of Customized Parts by Fused Deposition Modeling (FDM). Application to a Shoe Heel

**DOI:** 10.3390/polym12092119

**Published:** 2020-09-17

**Authors:** Amabel García-Dominguez, Juan Claver, Miguel A. Sebastián

**Affiliations:** Department of Manufacturing Engineering, Universidad Nacional de Educación a Distancia (UNED), 28040 Madrid, Spain; amabel.garcia@invi.uned.es (A.G.-D.); msebastian@ind.uned.es (M.A.S.)

**Keywords:** additive manufacturing, optimization, parametric design, infill optimization, mass customizing, biomechanics, FDM

## Abstract

Additive manufacturing technologies offer important new manufacturing possibilities, but its potential is so big that only with the support of other technologies can it really be exploited. In that sense, parametric design and design optimization tools appear as two appropriate complements for additive manufacturing. Synergies existing between these three technologies allow for integrated approaches to the design of customized and optimized products. While additive manufacturing makes it possible to materialize overly complex geometries, parametric design allows designs to be adapted to custom characteristics and optimization helps to choose the best solution according to the objectives. This work represents an application development of a previous work published in Polymers which exposed the general structure, operation and opportunities of a methodology that integrates these three technologies by using visual programming with Grasshopper. In this work, the different stages of the methodology and the way in which each one modifies the final design are exposed in detail, applying it to a case study: the design of a shoe heel for FDM—an interesting example both from the perspectives of ergonomic and mass customization. Programming, operation and results are exposed in detail showing the complexity, usefulness and potential of the methodology, with the aim of helping other researchers to develop proposals in this line.

## 1. Introduction

The opportunities and new possibilities offered by additive manufacturing are too wide to be exploited or explored only from approaches focused on the manufacturing process. The paradigm shift that these technologies allow is not only based on new manufacturing technologies and processes [1,2,3,4], but on the possibility of proceeding with design in a different way [5], as well as proposing different solutions from the traditional technologies for many products and designs. 

The limitations of the manufacturing technologies available to materialize a product determine its design. So, it would not make sense to have a set of new manufacturing technologies [6] which offer new possibilities, and continue considering previous design solutions that are conditioned by the limitations of other manufacturing technologies. It would mean obtaining the same result, but by using other techniques and without taking advantage of their possibilities, since reducing the limitations caused by new technology increases the ability of designers to offer new solutions previously impossible. Thus, it is reasonable to think that the progressive introduction of additive manufacturing technologies in the different productive sectors will mean the appearance of new design solutions to old problems in the coming years. In this context, it is necessary to explore new strategies to maximize capacity, synergies and the integration of new technologies such as additive manufacturing, parametric design and optimization, an objective to which the authors believe this work can contribute.

The high level of geometric freedom that additive manufacturing technologies allow [7] significantly increases possible design solutions. In this context, parametric design and optimization processes become very important [8]. 

Parametric design makes it possible to adapt a basic design to specific characteristics of use by modifying the value of some parameters. These design tools can be applied in any field, but their potential is especially significant in the case of products that must be adapted to the particular characteristics of each user, an approach of high interest for mass customization strategies. Thus, any product affected by ergonomic aspects can benefit from this type of approach. Commercial applications [9,10,11,12] in markets such as fashion or customized purchases are examples of this potential, but applications in the field of medicine can be of special relevance. More specifically, application of the proposed methodology to the case study developed in this work is considered of interest to channel the response to very different problems studied in the field of biomechanics, which could benefit from design strategies that incorporate some degree of customization [13,14,15]. On the other hand, when the number of feasible solutions that meet design objectives, such as lightweight parts, is high, optimization processes become very important as tools to guide the choice of the most appropriate solutions. These tools allow for the design and manufacture of optimized and customized lightweight structures in different fields and devices, such as customized neck orthosis [16] or load-bearing implants [17].

In any case, currently, additive manufacturing technologies also have some limitations. The standardization of some processes and products is an example [18,19]. Also, the materialization of the pieces itself presents some limitations, with special attention to the effects of process parameters [20,21,22,23,24,25,26,27], a topic widely studied in the scientific literature.

This work presents part of the results obtained in the research developed in the first author’s doctoral thesis [28]. Thus, the operation of a methodology designed to integrate additive manufacturing, parametric design and optimization within a continuous workflow is shown. This approach allows for obtaining optimized and custom designs to be manufactured using additive manufacturing. The structure of the methodology, its main parts and its operating logic have been studied in previous works [28,29]. However, the complexity and extension of the visual programming developed with Grasshoppers [30] requires a detailed exposition focused on the description of the coded parametric design and the way to connect them to link the sequence of operations and plug-ins [31] that define the final design. In this work, the application of the proposed methodology to a practical case allows for a better understanding of particular aspects, since the structure of the associated programming and the successive results obtained throughout the phases of the methodology are shown step by step.

The development of this work seeks to illustrate the potential and utility that the methodology can have in different fields, as well as to explain why the choice of the practical case was very thoughtful. In this way, a shoe heel was selected as a case study due to its simplicity, the need to meet clear mechanical requirements, the interest of part lightening through topology optimization and the need to adapt the design to the particular characteristics of each user. This last aspect is relevant because it makes it a good example for the implementation of the methodology in mass customization strategies [32], and it is an approach with which the authors are working [28,33].

The optimization strategy proposed in this work pays special attention to the optimization of the filling. The ability to define infill structures against the solid parts of traditional designs represents one of the most interesting advantages that additive manufacturing technologies offer. In this sense, the topology optimization of these structures in a heterogeneous way throughout the piece represents one of the main objectives of this study, allowing us to adapt the infill design to different load cases applied to the piece [34].

The step-by-step explanation of the operation of each part of the methodology and the effects that each part of its programming causes, is considered interesting and useful for the development of other similar design strategies in the future. The abundant graphic information that accompanies the work is also considered very useful since it illustrates the structure of the visual programming developed and the effects of its different parts throughout the design optimization process, a difficult task without directly using the software.

## 2. Materials and Methods 

The methodology’s objective is to design optimized lightweight parts while ensuring a good mechanical behaviour in a continuous workflow in order to be compatible with mass customization strategies. The lightening of the pieces is achieved by a topology optimization, a further structural optimization of its lattice infill and its wireframed shell, and finally, by a multi-objective optimization of the assembly in a continuous workflow. The continuous workflow allows the methodology to establish any part of the parametric design as a variable or objective in a continuous data flow in order to automatically generate the most suitable solutions without redefining the model in intermediate phases [29]. 

The proposed methodology is applied to the design of a shoe heel for a specific individual under a mass customization strategy. In this case, the most suitable methodological approach is the continuous methodology [29] since few variables are involved in the optimization problem and can be easily delimited in a single algorithm. On the other hand, the objective of the present work is to generate an optimized design that unifies infill and shell in a unique lattice structure of variable density, so it is not convenient to break the problem down into parts, but it is interesting to consider all the possibilities in the same iteration and in the same optimization problem.

### 2.1. Initial Design Considerations

The case study focuses on urban footwear—specifically, on its heel. No distinction is made as to whether there is a male or female user, since their morphological differences consist of the size of the foot and the weight of the person, as well as their loads’ distribution. This information can be considered in a personalized way with the scanning of the user’s feet and with the user’s gait analysis.

For the design of the last and the heel, the user’s foot, as well as its biomechanics are considered. Its scientific bases and recommendations, along with the size of the feet are considered.

#### 2.1.1. Biomechanical Considerations and Gait Analysis

Several phases are distinguished in the gait cycle [35,36], so in order to determine the loads to be supported by the shoe heel, the load distribution values of the hindfoot in the dynamic state of the gait analysis provided by the user are taken, since it is in the single support phase of the gait cycle when all the user’s weight falls on one foot and therefore the most adverse load case occurs. 

In the proposed case study, the right foot heel is designed. However, the methodology is applicable to both feet in the same way, only the load, the supports and the 3D model of the scanned foot vary but when they are introduced into the design problem, the optimized design of the heel is automatically updated. 

The load values distributions are compared to those theorical values given by the gait analysis shown in Table 1 and then a correction factor is calculated and applied. Furthermore, the deviation of the body load distribution between the forefoot and the hindfoot according to the heel height is also calculated, considering the values provided by Witana et al. [37]. In such a way that, based on the heel height, the individual’s weight, in this case 77 kg, is distributed between the forefoot and the hindfoot with its correspondent correction factor. On the other hand, to determine the support surface of the foot, the bitmap images of the report of the static analysis are taken, as well as its pressure distribution as it represents its broadest state. 

#### 2.1.2. Design Criteria for the Last and Heel from a Scanned Foot Model

The anthropometric foot measurements and its morphology are essential for the design of a shoe last; however, it is also necessary to consider biomechanical aspects and functional corrections for specific footwear aesthetics. In this sense, a scan of the foot in a static position with all the weight on it is done to get the maximum widths and ensure that the foot last is not narrower than the foot in any phase of the gait cycle and so that there is consistency with the visualization of the results of the static analysis of the tread of the report provided.

**Figure 1:**
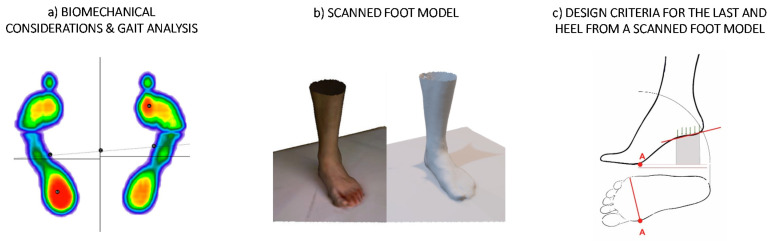
(**a**) Display of the gait analysis results [38]; (**b**) Scanned model of the foot at rest; (**c**) Outline of the most relevant aspects to consider in the design of the shoe last.

The most relevant aspects to consider in the design of the last’s seat are describes below and illustrated in Figure 1.

- Firstly, the head of the first and fifth metatarsals, which defines the width of the forefoot. This measure is critical for the adjustment of the footwear and in the terminal stance and pre-swing, the entire body rests in this place adopting its maximum width, which must be respected in the shape of the last.
- On the other hand, with regards to the heel, it is convenient that the individual’s weight rests on the shoe heel’s contact surface centre of gravity. Furthermore, it is highly recommended that the foot heel rests on a surface oriented with an angle whose value depends on the heel height, thus, the higher de heel height the greater de angle. Moreover, the heel height is considered without the sole, since it is also in the forepart, so only the difference in heights, between the horizontal and the support of the heel, is considered.
- Finally, the waist curve must be respected and adapted as far as possible to the curvature of the foot.

**Figure 2:**
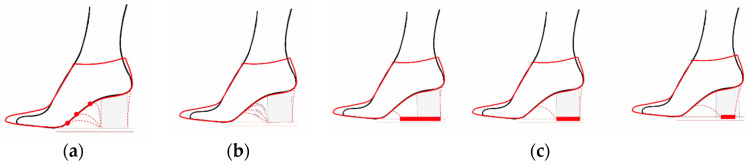
(**a**) Diagram of possible design variations when modifying the starting point of the heel; (**b**) Diagram of the possible design variations when modifying the heel curvature; (**c**) Diagram of possible design variations when modifying the heel support surface.

Regarding the design of the shoe heel, a series of design variables, shown in Figure 2, are established in addition to its height, and they modify its shape as it is generated from a parametric design.

- Firstly, the shoe heel seat starting design point is determined as a variable. It can be taken from the waist at any point, without exceeding the foot seat in any case.
- Secondly, another design variable is the curvature that the heel adopts in its shape. Even when it starts from the same point it can take different shapes depending on the tangencies that are established from the initial and final point.
- Finally, the heel support surface is considered as a variable. From the foot heel’s support surface taken from the gait analysis an equidistance of its boundary is generated in order to create different heel designs while maintaining its centre of gravity.

### 2.2. Initial Technological Considerations

In addition to considering specific aspects of the product and its design, it is necessary to consider aspects related to the technologies used in the process of optimizing the design and its subsequent manufacturing.

The proposed methodology and the programming structure designed are applicable to any additive manufacturing technology [28,29]. The sections and subsections defined through the programming face design problems that are approachable from the different additive manufacturing technologies, thanks to their common layer-by-layer forming strategy and the geometric freedom that this provides. However, it is necessary to consider aspects of influence and which are specific to each particular technology, and incorporate those aspects, and the values associated with them, into the methodology as input information. In this work, FDM was the selected manufacturing technology and Grasshopper was the selected coding language for the programming structure of the methodology proposed [39].

#### 2.2.1. Selection and Characterization of the 3D Printing Material

For the application of the methodology to the optimized design of a heel, ABS filament is taken due to its extensive use in FDM technologies. It is difficult to characterize the anisotropic properties of the material once printed. However, there are several experimental studies that attempt to offer values for the characterization of the mechanical properties of 3D printed ABS such as Cantrell’s study [40] from which most of the values for the characterization of the ABS are taken. Further experimental work has also been developed in previous works in order to characterize the density of printed parts [18,28]. 

#### 2.2.2. Selection of the Infill Structure Design

**Figure 3:**
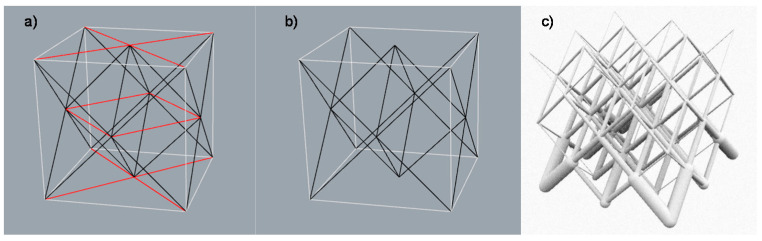
(**a**) Octet-truss unit cell structure; (**b**) Unit cell applied to the case of study; (**c**) Size optimization done to the resultant lattice structure.

Regarding the selection of the infill structure design, an open cellular structure is chosen in order to be able to remove any needed support material [41]. Furthermore, a lattice structure is defined due its stiffness [42] and an octet-truss unit cell design is taken as a starting point where only the linear elements at 45° are considered so that the structure is less conditioned by the printing direction, as shown in Figure 3. Further studies are developed in previous works to determine the dimensional tolerance to be applied on the nominal diameter of each bar [28]. Moreover, a uniform repetition pattern is defined throughout the heel’s infill and a size optimization is done to each bar of the lattice structure [43]. 

#### 2.2.3. Selection of the Coding Tools Used

The methodology is developed in Grasshopper programming language. Grasshopper is a visual coding application integrated in Rhinoceros (Robert McNeel and Associates, Seattle, WA, USA). The most appropriate plug-ins, shown in Table 2, are introduced, and applied in the methodology as helpful frameworks and algorithms for specific tasks. Furthermore, another coding language is also used in order to be able to code not only in the visual programming language of Grasshopper but also in the written programming language of Python. 

### 2.3. Workflow

**Figure 4:**
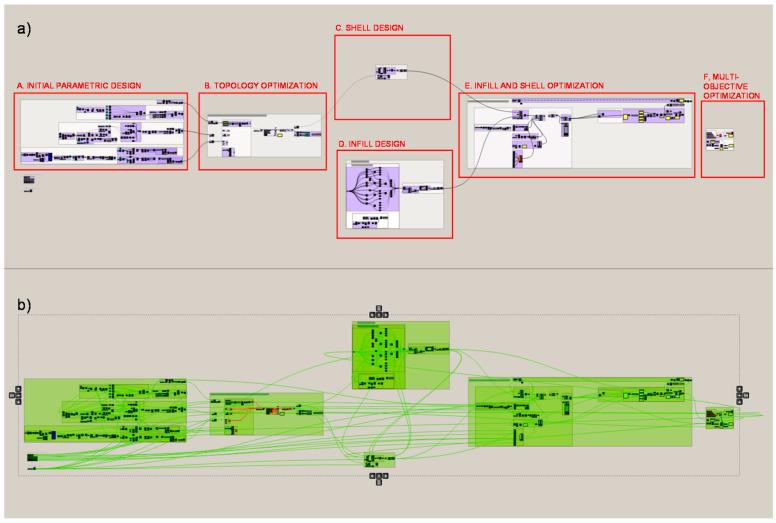
Programming structure definition of the proposed methodology for the case study developed with Grasshopper. (**a**) Structure diagram, (**b**) Data flow.

The workflow developed for the application of the methodology to the proposed case study is described below. The problem is divided into 6 sections and subsections in order to delve into the programming of the methodology. The programming structure definition of the proposed methodology for the case study developed with Grasshopper is shown in Figure 4. Firstly, the initial boundary of the heel is designed from a parametric approach. The geometric boundary is then topologically optimized and this final boundary is taken as a starting point for the shell and infill design of the piece. Finally, the shell and infill lattice structures are optimized from different approaches, first a size optimization and at last a multi-objective optimization that involves parameters from every part of the problem. The process and the result of each programming part is illustrated for a better understanding of the methodology’s workflow.

#### 2.3.1. Section A: The Parametric Design of the Initial Heel Volume

An initial heel volume is performed from a programmed parametric design structure. A series of variables configured from a range of viable values are involved in the design problem in order to be able to produce automatic alternative designs. On the other hand, those values and aspects that are given by the biomechanical determinations and from the anatomical and the gait analysis of the individual are considered constants.

**Figure 5:**
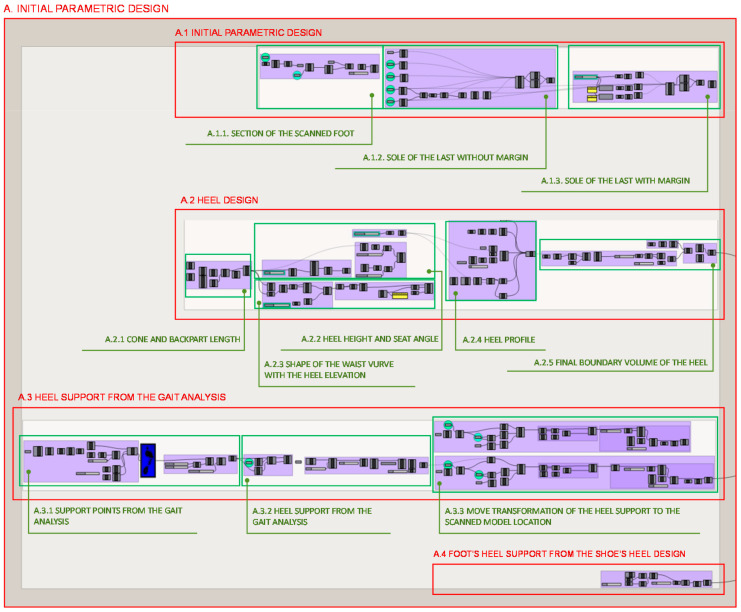
Programming structure definition of the initial parametric design of the heel volume.

The programming structure for the development of the parametric design of the initial volume of the heel consists of 4 parts illustrated in Figure 5 that take as a starting point the scanned model of the user’s foot, as well as the user’s gait study. In this way, referring the exposition to the alphanumeric designation of each part, the structure of each one, and of its subparts, is graphically illustrated and the fundamental aspects for its understanding are exposed.

The initial parametric design that defines de heel volume consist of 4 different subsections. Each one develops a different part involved in the design of the final heel volume. 

**Figure 6:**
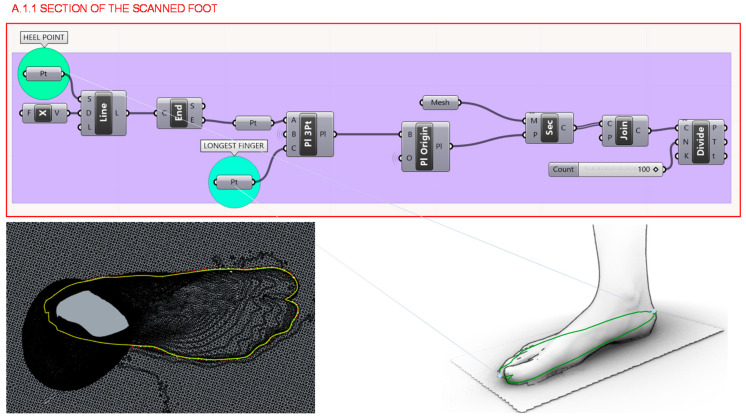
Programming structure definition to generate the section of the scanned foot model.

In section A.1. the design of the last’s insole takes the scanned model of the individual as a starting point and its programming structure consists of 3 other parts illustrated in Figure 5. Firstly, in section A.1.1. a section of the scanned foot 3D model is done by a plane defined by the two most prominent points of the foot: the heel and the toe, as Figure 6 shows. 

**Figure 7:**
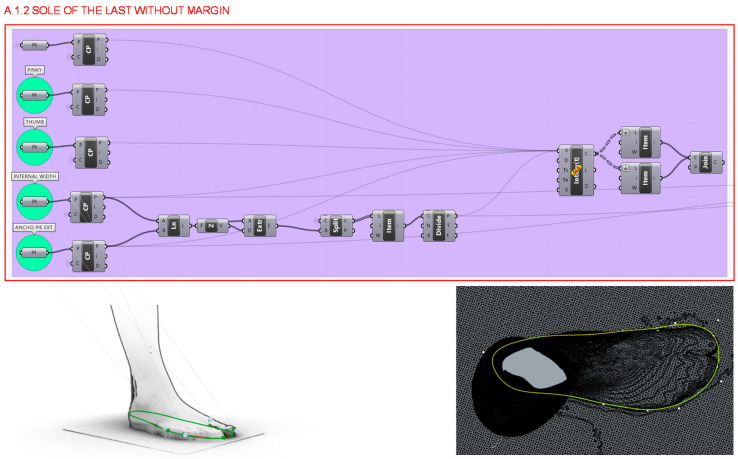
Programming structure definition to generate the sole of the last without gap.

From the previous step, in section A.1.2. the sole of the last without margins is designed by taking the previous curve divided into 100 sections and obtaining the closest segment’s points to the little finger, the thumb, and the first and fifth metatarsus as well as the most prominent finger. From these points curve is made to wrap around the sole of the foot without any gap. Figure 7 illustrates the coding developed as well as its results. 

**Figure 8:**
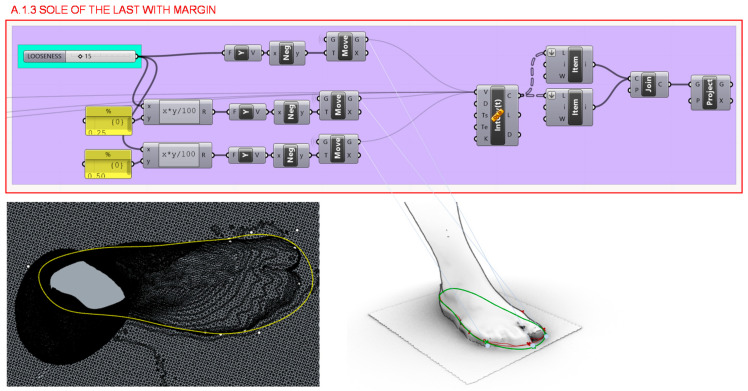
Programming structure definition to generate the sole of the last with margin.

Comfort margins of 15 mm are provided to the previous curve at 3 different points of the forefoot in section A.1.3., as shown in Figure 8. Although the margins are not given in a uniform way, a 25% is applied to the end of the little finger and 50% to the end of the thumb.

**Figure 9:**
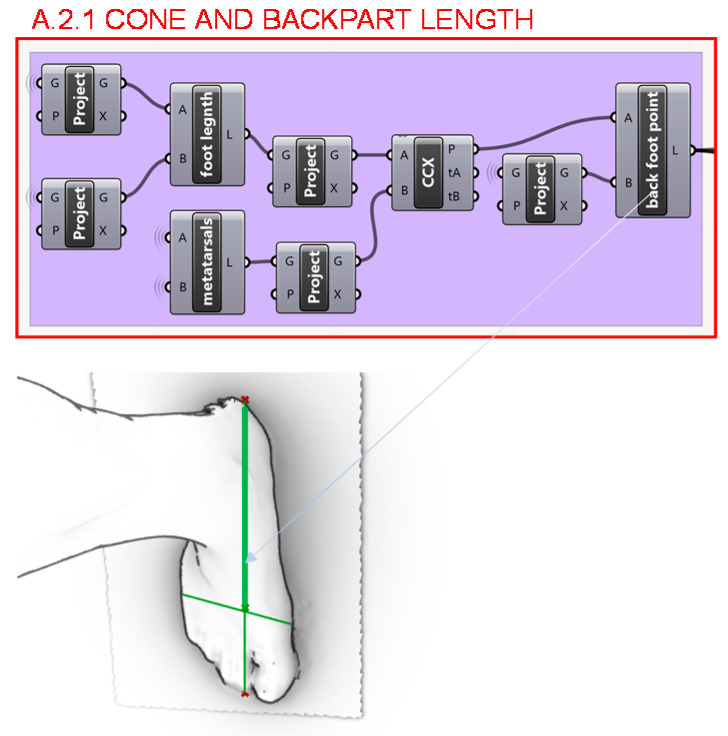
Programming structure definition to determine the length of waist and seat of the foot.

In section A.2. the heel design programming structure can be broken as well down into 5 parts, as it is illustrated in Figure 5. Firstly, in section A.2.1 the length of the waist and seat of the foot, which includes the midfoot and the heel, is calculated. It is found from the intersection between the longitudinal axis of the shoe last bottom and the line of the metatarsals, where the foot is flexed and where the weight in the swing phase of the gait cycle is supported, towards the back point of the last bottom. The visual programming of this step is shown in Figure 9.

**Figure 10:**
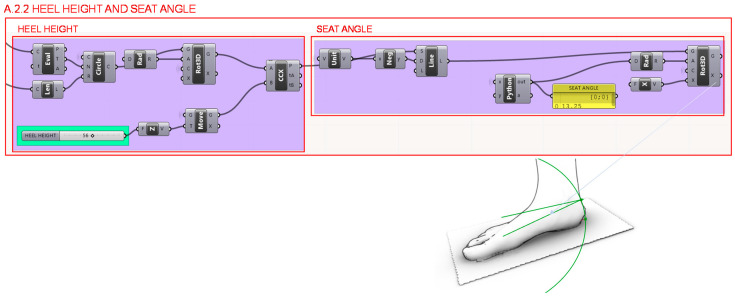
Programming structure definition to determine the heel height and its most suitable seat angle.

Furthermore, in section A.2.2. shown in Figure 10, the waist and seat profile are determined from the support point of the metatarsals depending on the height of the heel, simulating the process of a step. From the seat height the most suitable heel seat angle is determined for the chosen heel height, through a script programmed with Python and integrated in Grasshopper with Gh Python Script, and it is applied to the profile.

**Figure 11:**
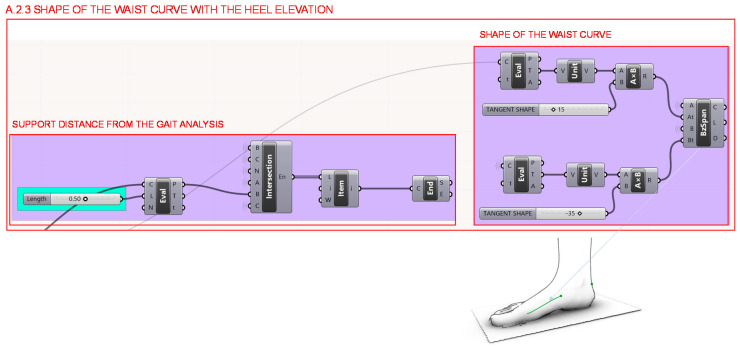
Programming structure definition developed to determine the shape of waist curve according to the chosen heel elevation.

Once the heel height and the forefoot support point have been determined, in section A.2.3. illustrated in Figure 11, the midfoot curve is developed, which should adjust as closely as possible to the shape of the foot. In order to shape the curvature as accurately as possible, the tangent vectors are taken at the point of the metatarsals and at the beginning of the last seat.

**Figure 12:**
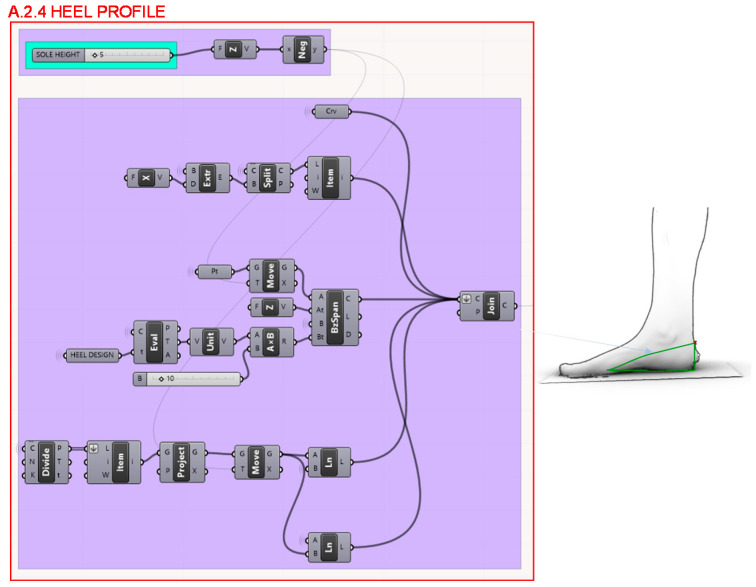
Programming structure definition developed to determine the heel profile.

Moreover, in section A.2.4., the waist curve blends with the last seat profile taking the same angle as the defined for its corresponding heel hight, as shown in Figure 12. In order not to restrict a priori the variety of possible design versions and optimize the geometry taking all the possibilities, the heel profile is left wedge-shaped. Different heel shapes are introduced as a variable to be optimized from the multi-objective algorithm, as described before, it depends on the starting point of the heel profile from the waist curve. 

**Figure 13:**
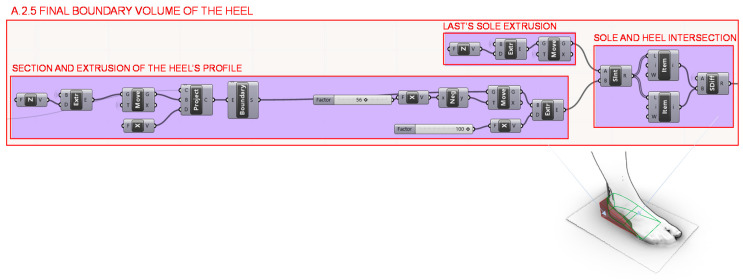
Programming structure definition developed to generate the final volume of the designed heel.

Finally, in section A.2.5., the final volume of the designed heel is defined from the Boolean difference between the extruded heel profile and the extruded sole of the last, each extrusion developed from perpendicular vectors to each other. The visual programming definition of section A.2.5 is shown in Figure 13.

**Figure 14:**
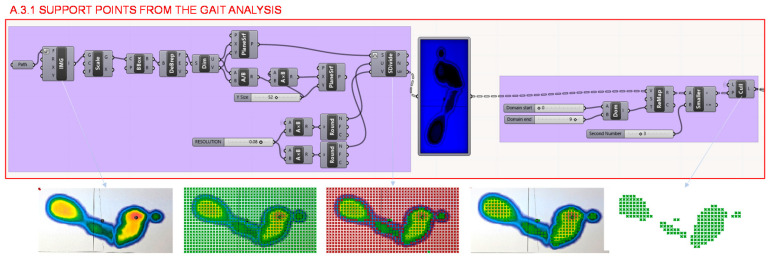
Programming structure definition developed to determine the support points of the foot from the gait analysis.

On the other hand, in section A.3., from the study of the user’s gait analysis, the heel seat is determined. The Grasshopper definition of the seat can be broken down into 3 parts as illustrated in Figure 5. Firstly, to determine the foot support area, in section A.3.1. illustrated in Figure 14, the image from the gait analysis is imported and from it the blue channel is extracted. A surface of the same size, proportion and location as the study image is decomposed in matrix of points. Each point analyses the colour component and an array of values is obtained, these values are then remapped in order to have control over the intermediate colour values so it is possible to take maximum pressure points or any point of foot support.

**Figure 15:**
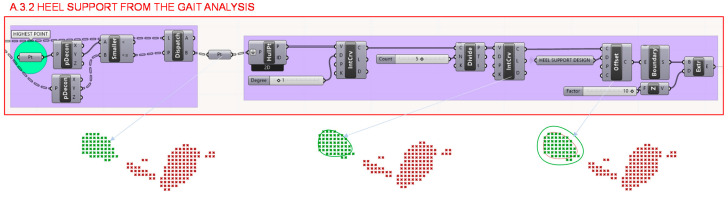
Programming structure definition developed to determine the support of the heel from the gait analysis.

In section A.3.2. the matrix of pressure points is then decomposed to take only those of the heel and from the externally located points a contour curve is generated that will define the geometric support of the shoe heel maintaining the same centre of gravity as the foot heel, following the indications of the Institute of Biomechanics of Valencia. In order to offer greater flexibility in the aesthetic characteristics of the design and to be able to offer thin or thick heels, an equidistance value is established for this contour curve, in such a way that it will always exist a correspondence in proportion and location with the foot heel, as well as the same centre of gravity. This step is illustrated in Figure 15.

**Figure 16:**
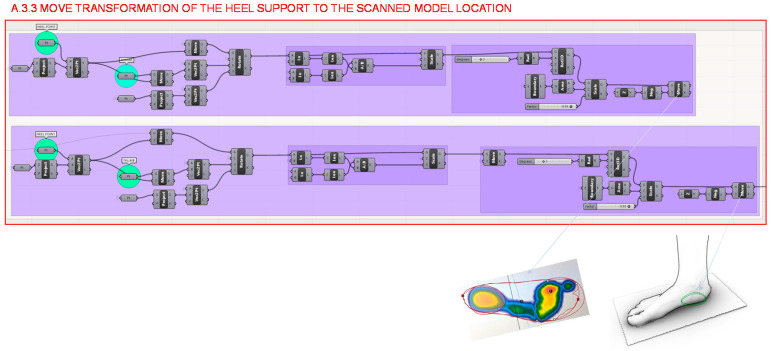
Programming structure definition developed to move the heel support to the location of the scanned model.

At last, in section A.3.3., the study carried out in section A.3.2 is translated to the location of the scanned foot model, matching the footprint from the gait analysis to the virtual model of the individual’s scanned foot, as shown in Figure 16.

**Figure 17:**
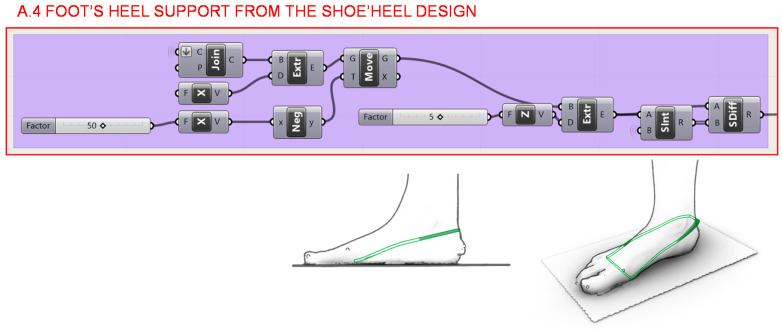
Programming structure definition developed to generate the foot seat on the volume of the designed heel.

Once the general volume of the wedge-shaped heel has been generated, in section A.4., the shape where the foot rests on is determined as shown in Figure 17. This geometry is taken as a premise in the Finite Element Analyses to determine the load locations in order to develop successive optimizations.

#### 2.3.2. Section B: Topology Optimization

Once the initial heel volume is generated, as well as the support of the heel from the parametric model, the topology optimization is done. The material is this way distributed in the most efficient way, attending to the load case determined by the gait analysis as well as by the biomechanical considerations. Millipede is used, it is a structural analysis and optimization plug-in that includes a Solid Isotropic Material with Penalization method (SIMP) algorithm for topology optimization.

**Figure 18:**
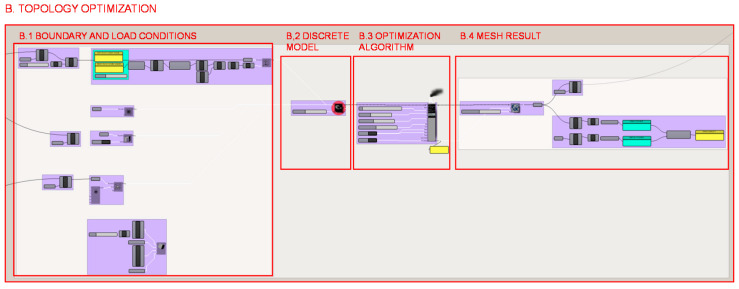
Programming structure definition developed for the topology optimization of the heel.

In the data structure developed for the topology optimization of the case study piece, 4 parts of the process can be distinguished, which are illustrated in Figure 18.

**Figure 19:**
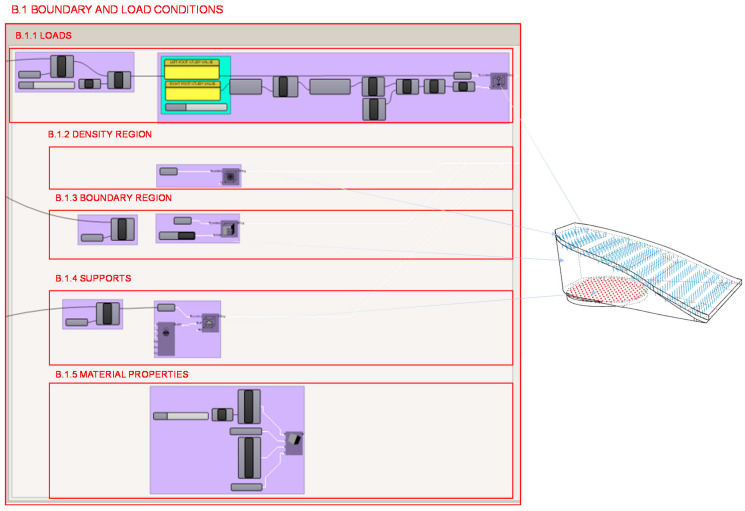
Programming structure definition developed for the boundary and load conditions for the topology optimization of the heel.

In section B.1., shown in Figure 19, the boundary and loading conditions are established. It is necessary to determine the location and value of the loads, the area of high material density, the starting volume, the location and type of supports and, finally, the mechanical properties of the material to be used.

The location of the foot heel load on the initial heel volume is determined in section B.1.1. from the foot seat shape developed in section A.4. The value of the load exerted on the heel of the right foot in the cycle of the step in which the whole individual’s weight rests on this foot is determined before with the biomechanical considerations and tread study. Consideration is given both to the distribution of the user’s weight in the most unfavourable case; this is in the single support gait cycle’s phase, and to the distribution of the weight between the forefoot and rearfoot with different heel heights. Both cases are calculated using integrated scripts in Grasshopper, either with mathematical expressions or with conditional structures programmed in Python.

**Figure 20:**
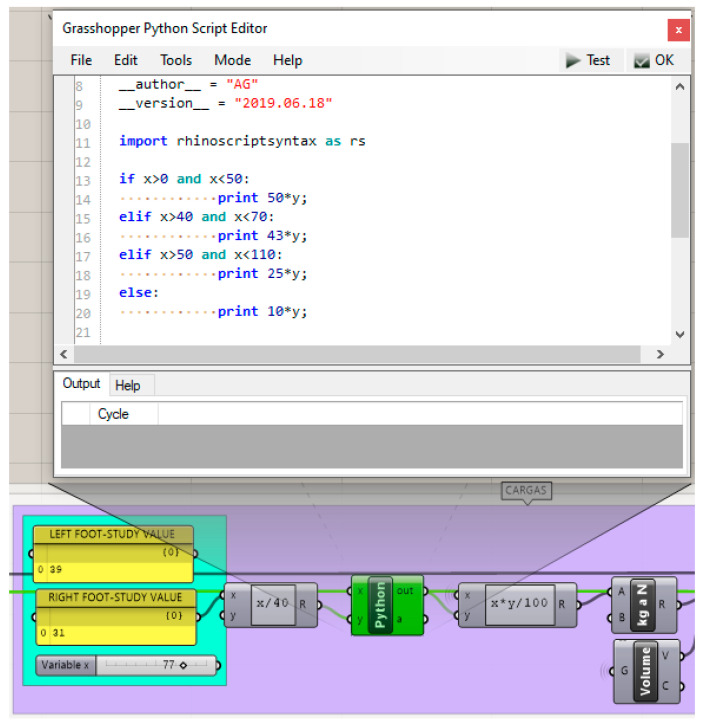
Programming structure for the calculation of the load distribution in the right hindfoot.

From the data obtained from the gait analysis and the values provided by Witana et al. [37], the load to be applied to the heel is obtained. These values are obtained using the programming structure shown in Figure 20 from the calculations previously explained.

It is necessary to define the boundary volume with a fixed density region in the footwear design to be able to assemble the heel with the other pieces. In this case, it is established in section B.1.2. as a condition to maintain the density of the seat geometry of the backpart developed in the initial heel volume design in section A.4. The boundary region is determined in section B.1.3. from the initial heel volume design to define the domain of the topology problem with the Millipede topological optimization algorithm. Fixed supports are defined in section B.1.4. as the support of the heel and its location are determined with the geometry generated in section A.3. from the individual’s gait analysis previously explained. The characteristic values of the material are introduced into the function in section B.1.5.

The Finite Element Model (FEM) is determined in section B.2. by introducing the resolution of the discrete meshed model as a variable. Depending on the entered value, a result will be achieved in greater or lesser detail. However, for greater detail, greater consumption of computer resources is needed. Therefore, it is necessary to find a balance between the detail to be obtained and the resources required, since this process, added to all of the data flow of the methodology, will be repeated a high number of times in the form of iterations to determine the most optimal result from the multi-objective optimization algorithm. In the case study developed for this work, low resolution values have been taken in order to reduce the resources needed, since the objective is not to develop a commercial piece but to check the feasibility of the methodology.

**Figure 21:**
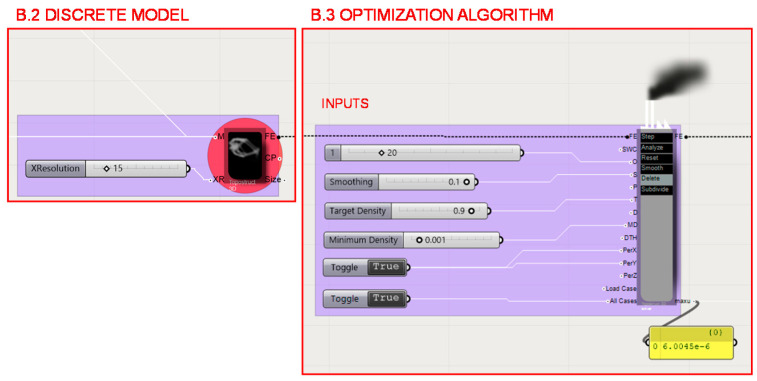
B.2. Programming structure definition of the discrete model for Finite Element Analysis (FEA) developed within the topology optimization algorithm. B.3. Millipede’s topology optimization algorithm and the introduced constants as inputs.

The inputs of Millipede’s topological optimization algorithm such as the number of iterations, the target density or the penalty factor are introduced in section B.3. as constants to simplify the process and not consume more computing resources. The programming structure definition of the discrete model for the FEA is shown in Figure 21.

**Figure 22:**
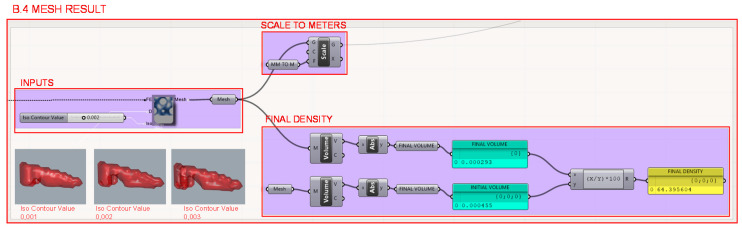
Programming structure definition of the resulting mesh from the topology optimization and its resulting volume percentage with respect to the initial volume.

Finally, in section B.4., the resulting mesh from the topology optimization is generated. The iso contour input value is entered as a constant; however, this value could be defined as a variable instead, since different values generate different geometries as illustrated in Figure 22. The resulting volume percentage with respect to the initial volume is then determined to be able to calculate the volume reduction produced by the topology optimization algorithm.

#### 2.3.3. Section C: Shell Design

**Figure 23:**
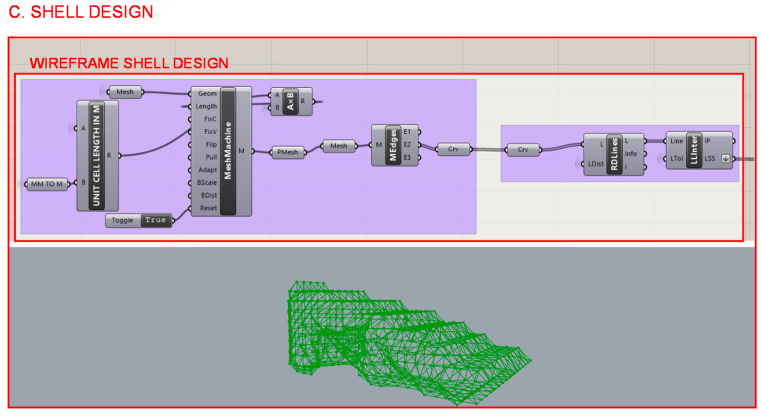
Programming structure definition of the shell design and its visualization in the Rhinoceros interface.

Based on the resultant mesh from the topology optimization algorithm, the design of the heel’s shell is defined as shown in Figure 23. In order to continue to lighten the piece, a wireframe structure with variable bar sections is selected to work collaboratively with the lattice infill structure. The lengths of the bars are adjusted to the size of the unit cell of the infill, establishing a mathematical relationship to reduce or enlarge them proportionally according to a scale factor. This is defined as a variable in the multi-objective optimization algorithm to find the best surface design for the proposed case study.

#### 2.3.4. Section D: Infill Design

**Figure 24:**
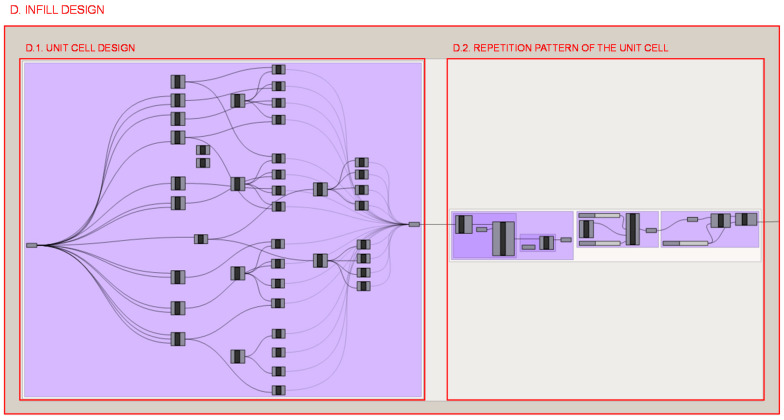
Programming structure definition of the infill geometry.

On the other hand, and based on the mesh solution generated by the topology optimization algorithm, the lattice infill design is defined. In the programming structure definition of the heel infill, 2 parts of the process can be distinguished and are illustrated in Figure 24.

**Figure 25:**
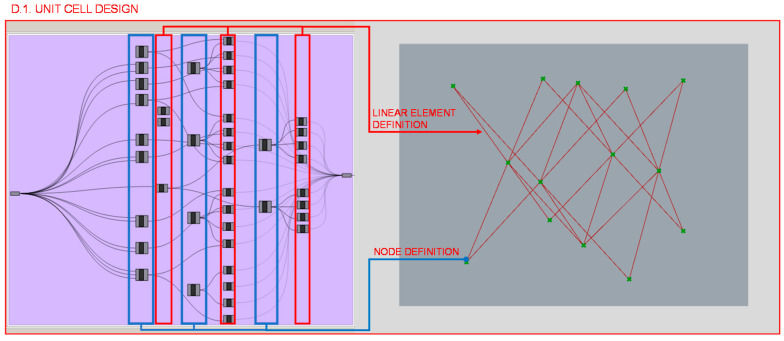
Programming structure definition of the infill unit cell and its visualization in the Rhinoceros interface.

Firstly, in section D.1., the unit cell design is defined as illustrated in Figure 25; a design similar to that of the octet-truss is chosen, although only the bars at 45° are taken and those that have other orientations are disregarded. Mathematical relationships are established between the coordinates of the points that define the endpoints of the linear elements so that the value of the unit cell size can be taken as the variable to be optimized.

**Figure 26:**
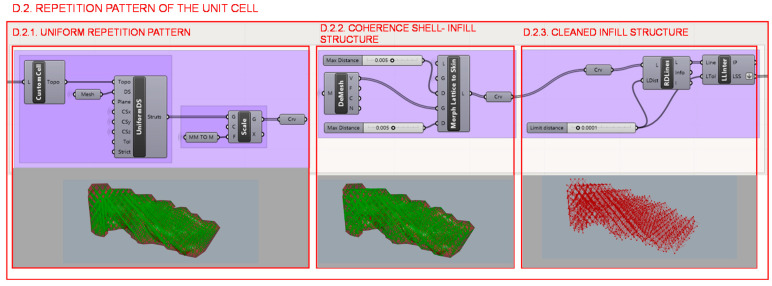
Programming structure definition of the infill pattern repetition and its visualization in the Rhinoceros interface.

Once the unit cell has been defined, in section D.2., it is repeated throughout the part according to the universal coordinate system and in a uniform and constant way as shown in Figure 26. The Intralattice plug-in algorithm [44] performs repetition and trims the linear elements that protrude from the part, while the Crystallon [45] algorithm performs the translation of the infill nodes of the elements that were cut by the surface contour and then moves to the location of the closest wireframe shell nodes. The maximum distance to be considered is restricted so that it only affects the closest shell nodes. Finally, to avoid duplicate elements or inconsistencies at node locations, a clean function is applied.

#### 2.3.5. Section E: Infill and Shell Optimization

**Figure 27:**
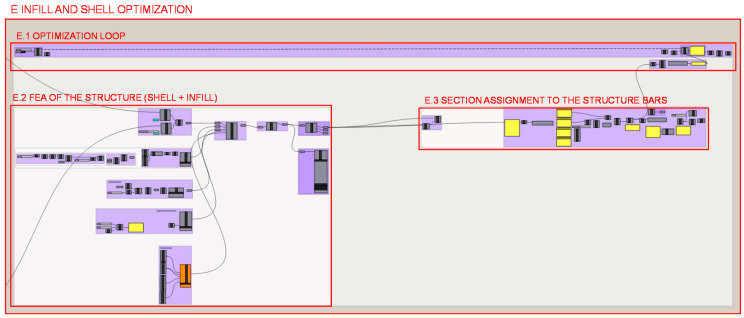
Programming structure definition of the structural optimization of heel lattice infill and wireframe shell.

A structural size optimization is developed, both of the wireframe shell and of the infill together since the structure works unitarily against the heel load conditions. Therefore, a lattice structure of variable density is automatically generated according to the bar stresses for the given load case. Figure 27 shows the different parts into which the programming structure definition of the structural optimization of the lattice structure of the infill and shell can be conceptually decomposed. 

**Figure 28:**
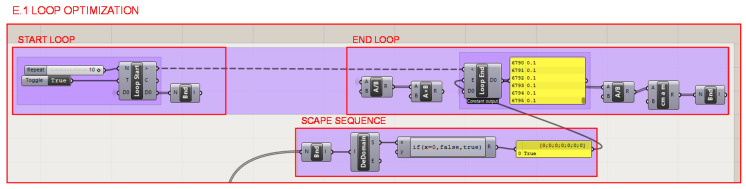
Programming structure definition for the generation of the structural optimization loop of the heel infill and shell.

The programming of a loop that iterates over the programming structures of sections E.2 and E.3 is developed in section E.1. and illustrated in Figure 28 in order to optimize the cellular structure in each roaming. Each iteration checks one by one, and through a matrix of values, if each bar mechanically resists the efforts to which it is subjected or not. In the case of compliance, it maintains its section, initially defined as the minimum the additive fabrication technology can manufacture, and in the case of non-compliance, it increases its section. A loop escape sequence is created to stop the iterations. A comparison and a conditional structure determines whether the normal forces of each bar exceeds the elastic limit of the material, even considering a safety factor.

**Figure 29:**
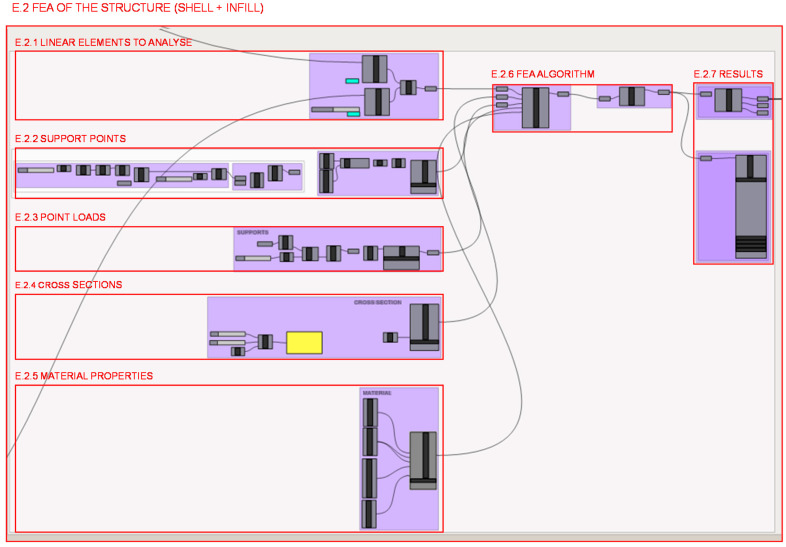
Programming structure definition developed for the FEA of the lattice structure of the heel infill and shell.

Figure 29 illustrates the different parts of the programming structure definition of the FEA carried out with the Karamba 3D plug-in [46] in section E.2. It illustrates the different parts defined by previous geometries and the values calculated for load and material characterization that are used as inputs to the load case for the FEA algorithm; therefore, no further zooms are made to the programming structure, as it is considered unnecessary for explanation of programming. The structural analysis is done for the lattice structure formed by infill and shell elements weld together in a unique structure. 

Firstly, in section E.2.1., the structural elements generated for the wireframe shell and those generated for the infill are joined in a unitary structure to be analyzed. Secondly, in section E.2.2., loads are configured, both their location and their value. In the first place, the position of the nodes that are included in the volume occupied by the geometry generated in section A.4 is determined. The coordinates of those points located in the same position as the foot seat on the heel are taken, as well as the load values considered for the topology optimization load case. On the other hand, the point supports of the heel are defined in section E.2.3., both their location and mobility restrictions. Those nodes in contact with the horizontal plane are taken, dispatching those whose z coordinates are greater than 0, with an error margin of 0.005 mm. On the other hand, the degrees of freedom of the fixed supports are defined.

The section of the bars of solid circular cross section is defined in section E.2.4. with the minimum diameter that the additive technology to be used—in this case FDM—admits, this is 1 mm as a starting point. It is defined independently for each bar by a matrix definition in order to be able to individually modify the dimension of each section depending on the needs. Besides, the material to be used is characterized in section E.2.5. with the same mechanical properties that were established for the topology optimization. Furthermore, the elements described in sections E.2.1., E.2.2., E.2.3., E.2.4 and E.2.5 are introduced in section E.2.6. as inputs to generate the FEM and then perform the FEA with the algorithm from the Karamba plug-in. The plug-in returns the numerical results of the stresses and moments to which the bars are subjected. Therefore, the results of the normals of each bar are extracted individually in section E.2.7. to analyze one by one whether or not they exceed the elastic limit of the material with a certain safety coefficient.

**Figure 30:**
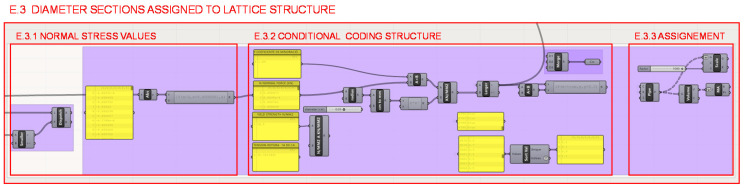
Programming structure for assigning the sections to the lattice structure.

In section E.3., illustrated in Figure 30, the resizing of the structure is carried out evaluating whether or not each bar exceeds the elastic limit, based on the values of the normals derived from the FEA. Next, a conditional programming structure is established, and finally, radius values for the sections are reassigned if necessary.

**Figure 31:**
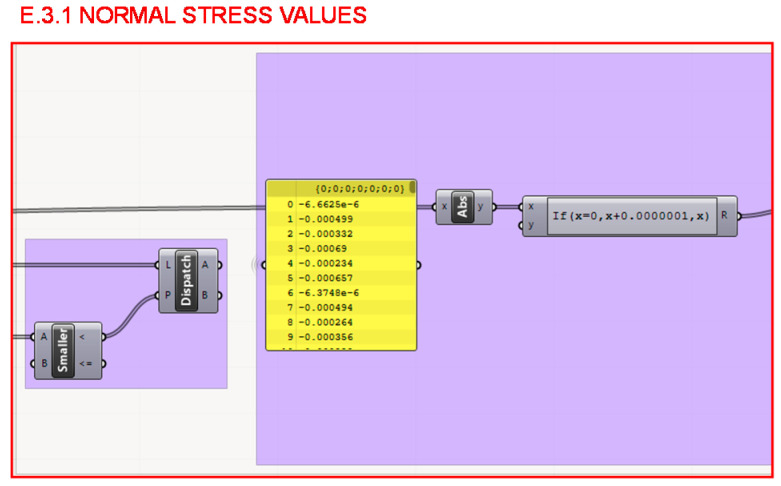
Programming structure definition to extract the values of the normals of the bars in absolute value.

**Figure 32:**
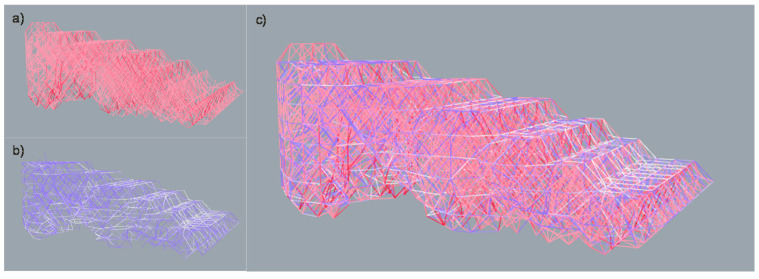
Visualization of the results of the FEA. (**a**) Bars subjected to compression; (**b**) Bars subjected to tension; (**c**) the whole of the structure with colour differentiation between the bars subjected to tension and compression.

Firstly, in section E.3.1. represented in Figure 31, all values are taken in absolute value so as not to differentiate between the bars subjected to tension or compression. A distinction could have been made between both; as shown in Figure 32, the determination was made to take the elastic limit of the material from the most unfavorable situation that is of tension and apply it to the entire structure for greater rigidity of the structure.

Moreover, in section E.3.2. it is verified that the tension to which each bar is subjected, considering a safety coefficient of 1.05, does not exceed the elastic limit of the material by performing the following calculation for each bar:

ƒ_y_ ≥ (N · γ)/πr^2^ (1)

where the tension to which each bar is subjected with a safety coefficient is represented by ƒ_y_, ABS elastic limit by N, the value of the normal of each bar represented by γ is the safety coefficient of 1.05 and r represents the circular cross section radius for the bar.

**Figure 33:**
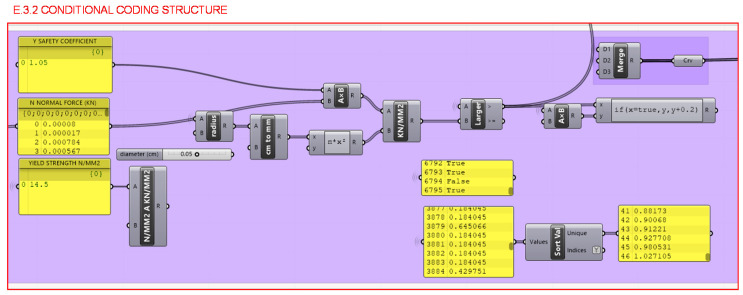
Programming structure definition of the comparison and conditional sequence to check the stiffness of the bars and resizing the bars in the event of being below the elastic limit.

A comparison and conditional structure are defined as shown in Figure 33; if the calculated value is less than the elastic limit of the material, a value of 0.2 mm would be added to the radius of the circular cross section of the bar. This process, together with the FEA, would be repeated as many times as necessary so that all the elements meet the requirement of having a lower value than the elastic limit of the material, thus ensuring the rigidity of the piece.

**Figure 34:**
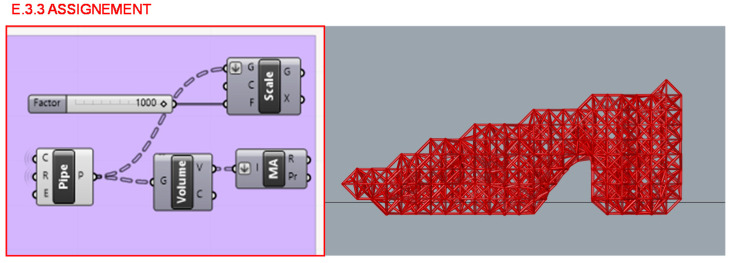
(**a**) Programming structure definition of diameter assignment to the circular cross sections of the structure bars calculated in step E.3.2. (**b**) Preview of the lattice structure result in the Rhinoceros interface.

Consequently, in section E.3.3. that is represented in Figure 34, the sections of the bars would gradually increase until every element of the structure resists the established load case. Therefore, the structure consists of a variable density lattice structure with a minimum cross section diameter of 1 mm.

#### 2.3.6. Section F: Multi-Objective Optimization of the Heel Design

The methodology is applied to the case study of the heel design with a continuous methodological approach; this is with a single multi-objective optimization algorithm at the end of the workflow, and after the lattice infill and shell optimization, as well as the topology optimization.

**Figure 35:**
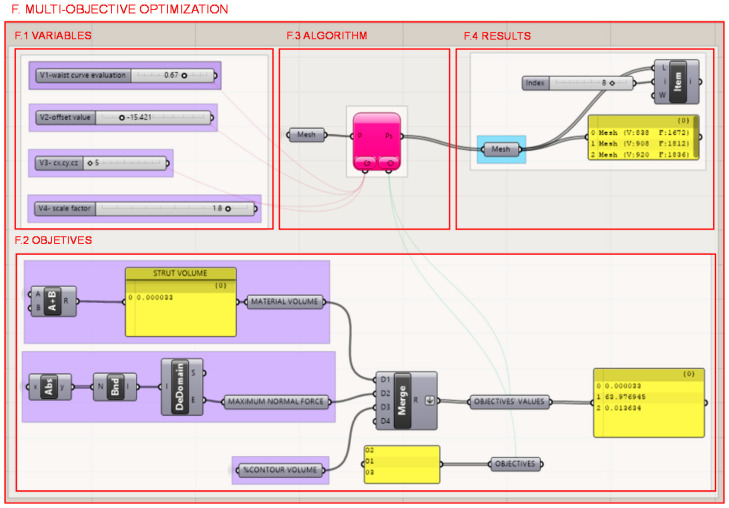
Programming structure definition to define the multi-objective optimization problem to be solved with the Octopus plug-in algorithm.

The optimization problem takes variables from different parts of the methodology data flow: from the starting heel volume design, the unit cell design of the infill and the shell design. Likewise, it takes as objectives data obtained in different parts of the process: the volume of the topologically optimized part, the volume of the lattice structure of the infill and the shell weld together into a single structure and the normal stresses of the bars. The programming structure definition of the multi-objective optimization problem to be solved in Octopus is illustrated in Figure 35.

**Figure 36:**
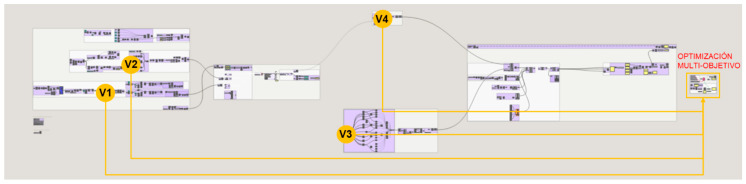
Location of the variables considered in the multi-objective optimization problem within the programming structure definition of the methodology for the case study.

On the one hand, in section F.1., the variables that take different values in the iterations produced by the multi-objective optimization algorithm are the following, and the location within the programming structure definition of the methodology for the case study is represented in Figure 36.

- V1—The advance of the heel in the waist path from the evaluation of the curve according to a range of values in which the value 0 corresponds to the beginning of the curve and the value 1 corresponds to the end.
- V2—The heel support surface defined as the equidistance to the heel seat surface extracted from the gait analysis of the individual described in section A.3.2.
- V3—The infill’s unit cell size where the three axes have their dimensions restricted to be equal values, introduced in section D.1.
- V4—The maximum length of the wireframe shell bars defined from a mathematical relationship with the unit cell size of the fill from a scale factor, its introduction is described in section C.

The defined variables take a number of finite values within a range of predefined boolean and float numbers that act as constraints in the search for feasible solutions.

- The infill cell size varies from 5 mm to 15 mm with an increase of 1 mm in its three axes alike.
- The advance of the heel design along the waist curve varies from 0 to 100% with an increase of 0.01%, where 0% corresponds to a wedge as it starts from the beginning of the waist path, and 100% corresponds to a narrow heel as it starts from the end of the waist path, next to the heel seat.
- The width of the heel determined by the offset value from the contour curve of the heel support surface extracted from the gait analysis. It takes varies from a negative value of 25 mm to a positive value of 25 mm with a step of 0.001 mm.
- The size of the wireframe shell bars, determined by a scale factor with respect to the infill’s unit cell size that configures the infill. This scale factor varies in a range of values from 0.5 to 2 with a step of 0.1.

The constraints established through the listed values are determined by biomechanical design criteria, as well as by size limitations of the manufacturing technology considered. 

**Figure 37:**
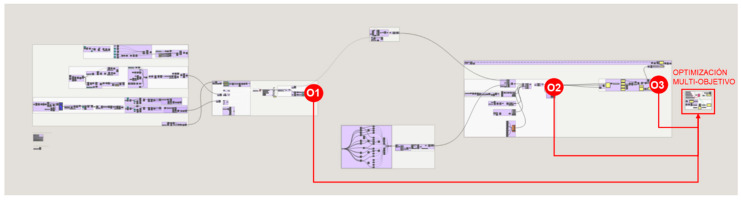
Location of the objectives of the multi-objective optimization problem within the programming structure definition of the methodology for the case study.

On the other hand, the defined objectives are defined in section F.2. and its location within the programming structure definition of the methodology for the case study is represented in Figure 37.

- O1—Minimize the percentage of volume of the result from the topology optimization with respect to the starting volume of the initial heel volume design.
- O2—Minimize the maximum normal stress of the lattice structure bars from the optimized structure conformed by the infill and the shell.
- O3—Minimize the volume of the optimized lattice structure conformed by the infill and the shell.

For the multi-objective optimization within the continuous methodology, the plug-in Octopus is used in section F.3. Octopus incorporates a multi-objective optimization evolutionary algorithm based on Pareto. These algorithms perform iterations modifying the values of the variables to obtain different solutions and thus generate populations. Each population of solutions is analyzed based on its achieved objectives, and identifies those located on the Pareto Front to simplify the search for the designer [47].

In Octopus plug-in various algorithms are available and for the resolution of the problem, the evolutionary algorithm SPEA-2 of Zitzler was chosen [48]. It is possible to guide the search for solutions by approaching the new generations generated to a selected individual solution, in such a way that the successive solutions will approach the objectives set in said selection.

The Octopus interface allows for the results to be visualized so that the designer can assess the results in a more intuitive way to be able to intervene and guide the search with greater criteria. This allows the morphology of the design to be visualized, as well as the values of the objectives achieved by each solution. 

The interaction of the designer or analyst in the iterative process of searching for optimal solutions is possible by directing the search from the visualization of geometric results, variables and objectives. Moreover, it is also possible because of the generation of solutions from a continuous data flow in the methodology. 

A resultant mesh is generated in section F.4. from the chosen element of the Pareto ready to export to a compatible format for an additive manufacture technology; FDM in this case. The selection of the final optimized design and the criteria considered are exposed below.

## 3. Results and Discussion

As a result of the application of the methodology to the selected case study where topology, size and multi-objective optimizations are simultaneously developed in a continuous workflow, 10 generations of a population size of 180 solutions are obtained. Those solutions that are located on the Pareto front are displayed in Octopus interface. From them, 6 solutions that are considered a priori most suitable are selected considering not only the objectives achieved, but also aesthetic aspects of design and their objectives are compared. The variables from which each of the solutions start are also collected to assess the most appropriate solution.

**Figure 38:**
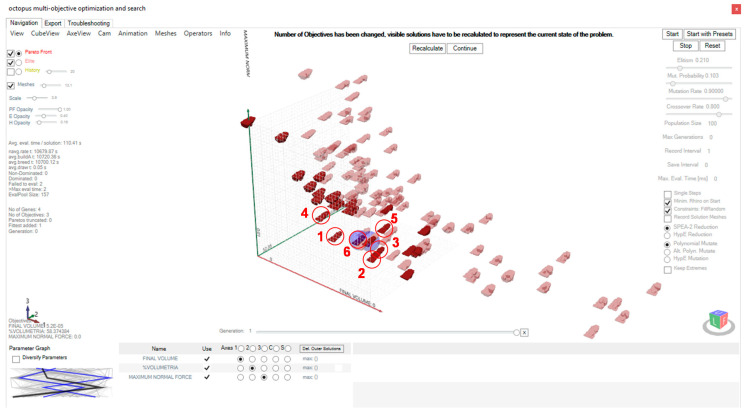
Selection of the 7 Pareto front solutions within the Octopus multi-objective optimization plug-in interface.

Figure 38 shows, in addition to the selection of the 6 solutions to be analyzed, the last population generated with the Pareto optima highlighted with an opacity of 100%. All of them are within the feasible region since in the definition of the problem the values of the variables were restricted to those possible for the manufacturing technology to be used. The mutation of the variables of the solutions achieved is also visualized in the parameter graph. Table 3 shows the infill and shell lattice structure of the generated heel, as well as their variables values and the achieved objectives. These values where extracted from the Octopus interface and from the recorded history of the iterations.

**Figure 39:**
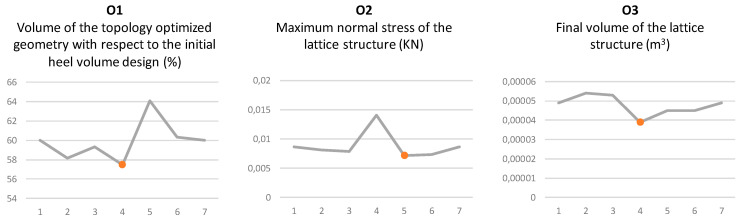
Comparison of the three objectives achieved by the 6 selected solutions, with the minimum reached marked red.

The objectives achieved by each of the solutions are compared in order to select the most suitable one, as shown in Figure 39. On the one hand, solution 4 achieves the lowest values of volume for the topology optimized geometry and material quantity for the final lattice structure solution. However, it achieves the highest value of normal maximum stress in its cellular structure. On the other hand, solution number 5 achieves the lowest value of maximum effort in the bars of its structure, although with the highest value of general volume.

Solution 4 has been selected as the most suitable since despite having a greater maximum normal force, and after the size optimization, it still has the smallest volume of the 6 compared solutions, which would mean material savings. On the other hand, since it has a lower general volume, it would also entail a lower cost since it would occupy a lower volume in the printer in order to manufacture together with other parts.

**Figure 40:**
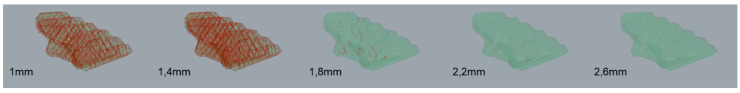
Visualization of the lattice structure decomposed by groups of bars with different diameters.

The diameters applied to the bars of solution number 4 in order to be able to resist all efforts are as follows: 1 mm, 1.4 mm, 1.8 mm, 2.2 mm and 2.6 mm. However, very few bars need to be resized into diameters above 1.4 mm as showed in Figure 40. 

**Figure 41:**
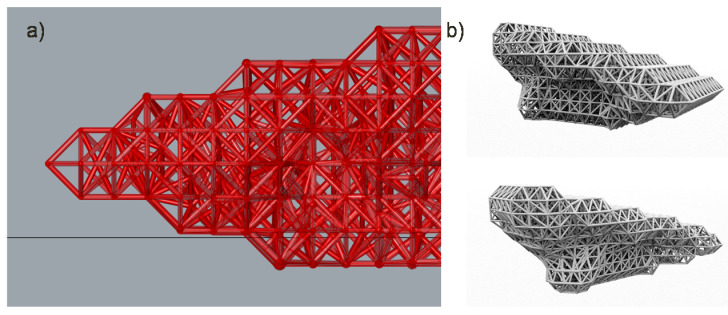
(**a**) Detail of the variable density lattice structure. (**b**) Visualization of the 3D model of solution number 4.

The increase of the diameter section applied to the bars is carried out gradually. In Figure 41 the variable density infill and shell lattice structure is represented. 

The result of the adopted solution number 4 is shown in Figure 41, where the heel rests on the boundary volume of fixed density defined from the topology optimization defined by a reduction of 20.31 mm from the foot heel seat extracted from the gait analysis. The initial volume design of the heel does not cover the entire waist curve profile, but starts from practically half to 42% of the path. Finally, in regard to the infill and shell size, solution 4 adopts a lattice infill unit cell size of 11mm in all its axes and the wireframe shell elements take a larger value where a factor of 1.6 scale is applied.

## 4. Conclusions

The application of the proposed methodology to the developed case study offers design results that are considered of interest and utility. A topologically optimized heel geometry has been obtained, but in addition, the infill and shell of the piece has also been optimized in the same process, adapting it to local forces in a non-continuous way throughout the piece. In this way, the methodology generates as a result a cellular structure that extends along the optimized geometry of the part which is composed of an open cellular infill structure and a wireframe shell. This structure is the result of a multi-objective optimization process and presents variable bar diameters throughout the part according to the existing stresses in each area of the part. The multi-objective optimization problem has been raised without formal restrictions and considering variables prior to topology optimization, which allows for avoiding a preconception of the design that can lead to less optimal solutions.

This approach is considered applicable to many other contexts. Other designs that require a certain degree of customization to adapt ergonomic aspects to the characteristics of the final user are good examples in which the application of this methodology can be useful. Biomechanical applications are therefore one of the lines of work that the authors want to explore in future developments derived from this work. But the application of the methodology is also operative as a support for the design of products of other types whose topology optimization needs to be addressed considering different objectives and acting on different variables. The optimization of structural parts or furniture would be examples of this.

The step-by-step explanation of the methodology, the level of detail and the graphic support of the work are considered useful as a reference for other researchers who are working on design strategies with approaches similar to that of this study.

## Figures and Tables

**Table 1:** Dynamic state load distribution values obtained with a gait analysis. [38].

Description	Values	Normal	Tolerance
Left forefoot load distribution	66%	60%	±3%
Left rear foot load distribution	34%	40%	±3%
Right forefoot load distribution	69%	60%	±3%
Right rear foot load distribution	31%	40%	±3%

**Table 2:** Selected plug-ins applied in the proposed methodology.

FEA	Topology Optimization	Multi-Objective Optimization	Lattice Infill	Loop
Karamba	Millipede	Octopus	Crystallon	Anemone
			Intralattice	

**Table 3:** Multi-objective optimization solutions with its variable values and achieved objectives. V1—Heel advance in the waist path (range from 0 to 1), V2—Offset value for the boundary of the heel support surface (mm), V3—lattice infill unit cell size (mm), V4—scale factor for the length of the wireframe shell elements, O1—Volume of the topology optimized geometry with respect to the initial heel volume design (%), O2—Maximum normal stress of the lattice structure (KN), O3—Final volume of the lattice structure (m^3^).

Solution	Variables	Objectives	Solution	Variables	Objectives
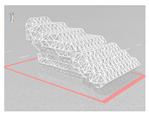	V1 = 0.09 V2 = 10.519 V3 = 9 V4 = 1.0	O1 = 60.000000 O2 = 0.008674 O3 = 0.000049	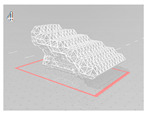	V1 = 0.09 V2 = 20.313 V3 = 11 V4 = 1.6	O1 = 57.468354 O2 = 0.014083 O3 = 0.000039
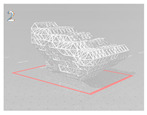	V1 = 0.66 V2 = 16.472 V3 = 7 V4 = 1.8	O1 = 58.166189 O2 = 0.008149 O3 = 0.000054	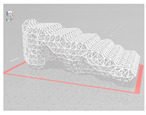	V1 = 0.01 V2 = −19.807 V3 = 10 V4 = 1.7	O1 = 64.096160 O2 = 0.007184 O3 = 0.000045
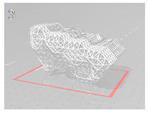	V1 = 0.09 V2 = 10.560 V3 = 7 V4 = 0.7	O1 = 59.327217 O2 = 0.00787 O3 = 0.000053	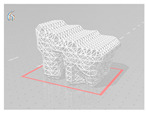	V1 = 0.97 V2 = −8.578 V3 = 8 V4 = 1.7	O1 = 60.338983 O2 = 0.007350 O3 = 0.000045
